# Spatial distribution and determinant factors of unmet need for family planning among all reproductive-age women in Ethiopia: a multi-level logistic regression modelling approach

**DOI:** 10.1186/s40834-022-00178-9

**Published:** 2022-08-01

**Authors:** Melkalem Mamuye Azanaw, Dawit Tefera Fentie, Yeaynmarnesh Asmare Bukayaw, Ayenew Molla Lakew, Malede Mequanent Sisay

**Affiliations:** 1 Department of Public Health, College of Medicine and Health Sciences, Debre Tabor University, Debre Tabor, Ethiopia; 2 Amhara Regional Health Bureaus, Zonal Health Office, Gondar, Ethiopia; 3 Amhara Regional Health Bureaus, University of Gondar Referral Hospital, Gondar, Ethiopia; 4 Department of Epidemiology and Biostatistics, Institute of Public Health, College of Medicine and Health Science, University of Gondar, Gondar, Ethiopia

**Keywords:** Unmet need, Women, Ethiopia, Spatial Analysis, Multi-level logistic analysis

## Abstract

**Background:**

Unmet need for family planning has been remaining high in developing countries than developed countries, notably in sub-Saharan Africa. Data on unmet needs can help countries set service priorities. This study aimed to explore the geographical disparities of unmet need among reproductive-age women in Ethiopia using a 2016 national population-based survey.

**Methods:**

This study was based on the nationally representative 2016 Ethiopian Demographic and Health Survey data. We used a total weighted sample of 15,683 reproductive-aged women. A multi-level logistic regression analysis was used to account for the Demographic Health Survey data’s hierarchal nature. In the multivariable multi-level analysis, those variables with a *p*-value < 0.05 were significantly associated with unmet needs. Spatial autocorrelation techniques were used to explore the clustering tendencies of unmet needss using Getis-Ord Gi* statistics.

**Results:**

Overall, 15.2% (95% Confidence Interval (CI): 14.63, 15.76) of women of the reproductive age group in Ethiopia had an unmet need for family planning in 2016. In multivariable multilevel logistic regression analysis; individual-level variables such as being married (Adjusted odds ratio (AOR) = 25.7,95% CI: 11.50,60.42), lowest wealth status (AOR = 1.43,95% CI:1.14,1.79), having five or more children (AOR = 1.98, 95% CI:1.62,2.41), being a follower of Muslim religion (AOR = 1.35,95% CI:1.03,1.76) and protestant religion (AOR = 0.73,95% CI: 0.53,0.99) than orthodox Christian followers were statistically associated factors with unmet need. Among community-level variables; being in rural residency (AOR = 1.37, 95% CI: 1.01, 1.93), belong to the Oromia region (AOR = 1.53, 95%CI: 1.10, 2.11) and Somali region (AOR = 0.37, 95% CI: 0.22, 0.61) were significantly associated unmet need. The spatial analysis of unmet need among all women revealed that Oromia, Southern Nations, and Nationality of People and Gambela regions had high hotspots than other parts of the country.

**Conclusions:**

In this study, the prevalence of unmet needs was high. Significant regional unmet need variation was indicated among reproductive-age women in Ethiopia, specifically in western parts of the country. Wealth status, number of children, marital status, residence, and religion were the most important associated factors with unmet needs. Addressing unmet needs targeted rural residents with low socioeconomic status, and western regions should be given top priority.

## Plain Language Summary

In general, the unmet need for family planning has been remaining high in developing countries, notably in sub-Saharan Africa. This study aimed to explore geographical disparities of unmet need for family planning among all reproductive-age women in Ethiopia using a 2016 national population-based survey.

We used a secondary data analysis of the 2016 Ethiopian demographic health survey. A total of 15,683 women aged 15–49 years were selected using a two-stage stratified sampling process.

Overall, the prevalence of unmet need for family planning was 15.2% (95% CI: 14.63, 15.76) in Ethiopia. The spatial analysis of the unmet need for family planning revealed that Northern and Western parts of Oromia, North of Southern Nations and Nationality of People and Gambela regions had high hotspots than the remaining parts of the country.

Generally, the findings indicate significant regional variation in the unmet need for family planning among reproductive-age women in Ethiopia, specifically in western parts of the country. Being in low wealth, having a higher number of living children, being in a Married or union relationship, living in rural areas, older age, being in Muslim and Protestant followers were found associated with unmet need for family planning.

## Background

Globally, unintended pregnancies have severe consequences for women’s health and their families, including high maternal mortality and unsafe abortion, particularly in developing countries. More than 358,000 women died due to pregnancy-related causes every year in the world [1, 2]. A study in 172 countries showed that modern contraceptives methods prevent more than 54 million unintended pregnancies, including 21 million unplanned births, 26 million abortions (of which 16 million would be unsafe), and seven million miscarriages; this would also prevent 79,000 maternal deaths and 1.1 million infant deaths [2]. Other studies indicated that a high fertility rate affects socio-economic development,,particularly among developing countries. In Ethiopia, the unmet need for family planning among all women dramatically declined from 25.3% in 2011 to 15.2% in 2016. On the other hand, satisfaction with modern contraceptive use increased from 50.7% in 2011 to 65.1% in 2016 [1, 3, 4].

Unmet need for family planning is defined as the proportion of fecund, sexually active women who want to limit or delay childbearing beyond two years but who are not using any method of contraception (modern + traditional methods); the sum of unmet need for spacing plus the unmet need for limiting births [5–7]. Globally, the unmet need for modern methods declined marginally from 15.1% in 2000 to 14.2% in 2019 [8]. The most significant declines are expected in Eastern Africa, where unmet need is projected to fall from 22% in 2017 to 16% in 2030 [9]. However, the unmet need has remained below 60% in Ethiopia with substantial regional variability. The prevalence ranged from 6.9% in the Somali region and 84.7% in the Amhara region [3, 10–12].

A study in different Asia countries showed that the unmet need for family planning had a positive association with having more children, women, having any media exposure, Muslim women, engaged in unskilled work, low level of education. However, it had a negative association with having one or two children [13–15].

A study done in Burkina Faso showed that significant determinants of unmet need for FP were having more children, being married more than once, decision-making on spending personal earnings, and women who desired fewer children. Other studies in Botswana, Sudan, and Ghana revealed that women and husband’s education, women’s occupation, low economic status, no history of parity, and being in the age group of 25–34 years were statistically significant factors to unmet need for family planning [16–20].

Studies in different Ethiopia regions showed that age, age at first marriage, educational status, religious factors, media exposure, discussion with a fieldworker, women health facility visit and discussion with a health worker at the health facility were associated factors with unmet need for family planning. Moreover, previous spatial studies at the national level showed regional variation in modern contraception prevalence rate where Addis Ababa, Amhara, and some parts of Gambela and Benishangul-Gumuz regions have a high contraceptive prevalence rate. Most of the high contraceptive prevalence clusters were located in the Addis Ababa region, while clusters of low prevalence were in Afar, Somali, and some parts of the Gambela region [10–12, 21–33]**.**

Unmet need for family planning is an important indicator used mainly for women’s reproductive health advocacy and the monitoring/evaluation of implemented programs incredibly Sustainable Developmental Goals (SDGs) [34]. Even though several studies on the unmet need for family planning in previous studies, most of them were taking only individual-level analysis by omitting the cluster effect. This study took into account those different analysis levels, including the spatial variation of unmet need for family planning across regions and identifying individual and community level predictors associated with unmet need for family planning among reproductive-age women in Ethiopia (EDHS 2016 dataset) 2022.

## Methods

### Study area and data source

The study was conducted in Ethiopia, located in the North-Eastern part of Africa, also known as Africa’s horn, between 3^0^ and 15^0^ North latitude and 33^0^ and 48^0^ East longitudes. Ethiopia is the 12^th^ most populous country globally, the second-most populous nation on the African continent (after Nigeria), and the most populous landlocked country in the world with over 109 million inhabitants as of 2019. The country has a total area of 1,100,000 square Km (420,000 square meters). Ethiopia is divided into ten ethnically based and politically autonomous regional states and two chartered cities (Addis Ababa and Dire Dawa). The regions are subdivided into sixty-eight zones and then further into 550 woredas and several special woredas.

In Ethiopia, healthcare service has been improved after implementing the Health Sector Development Plan through decentralisation into a three-tier structure. The primary health care unit mainly provides preventative and essential curative services with a referral system to the nearest high level of care. Primary healthcare, including family planning, is offered free of charge to all women. Health extension workers staff the health posts to improve the universal primary healthcare (PHC) coverage at the lowest administration level.

This study used the 2016 EDHS dataset, which was implemented by the Central Statistical Agency and was conducted by Federal Ministry of Health (FMoH) with technical assistance of ICF [35]. The Ethiopian DHS is a nationally representative survey conducted every five years to assess its health status. The Ethiopia DHS provides population and health-related indicators of the country and regions. Data were accessed from their URL: www.dhsprogram.com by contacting them through personal accounts after justifying the reason for requesting it. Then reviewing the account, permission was given via email [3].

### Study design, population, and sampling procedure

Secondary data analysis of the 2016 Ethiopia Demographic and Health Survey (EDHS 2016) was conducted. The EDHS employed a stratified two-stage sample design by identifying 645 (202 urban and 443 rural) enumeration areas or clusters identified by the 2007 Ethiopia Population census and sampled households from all eligible households within each cluster. The source population consists of all women of reproductive age in Ethiopia. The study population included women of reproductive age in the households located in the primary sampling units (PSUs) in the 645 Enumeration areas sampled in the first stage. The sampled population comprises women of reproductive age who live in a random sample of 18,008 households in Ethiopia [3]**.**

Respondents to the survey included all women aged 15–49 years within five years before the survey. A total of 15,683 women of the reproductive age group were included in the final analysis. Collected data include indicators of fertility, reproductive health, maternal and child health, mortality, nutrition, and self-reported health behaviour among adults. We obtained data on demographics and community level, Global Positioning System (GPS), and unmet need for family planning. Datasets were linked by cluster, house number, and line number. These were subsequently linked with the GPS data by cluster and region to compile the final data for analysis.

The outcome interest is an unmet need for family planning, and subjects were classified as (Yes/No). Women’s unmet need for family planning was measured by asking questions all women, whether they are currently using any form of contraceptives or not. Women who were currently not using any form of family planning were further asked whether they did not wish to become pregnant (unmet need for limiting) or within the next two years (unmet need for spacing). The independent variables were individual and community-level characteristics. The individual factors included age, religion, marital status, educational status, number of children with current pregnancy, age at first marriage, knowledge of any methods, wealth index, visited by family planning workers, visited a health facility, and exposure to mass media whereas the community-level factors were residence (urban and rural), region, community women education & community media exposure. The GPS coordinates for each respondent were included in our analysis to represent the clustering of subjects graphically. The DHS randomly displaced these coordinates by 5 km to protect the confidentiality of respondents. About 21 clusters were excluded due to having 0 coordinates.

### Statistical analysis

The analysis was conducted after sample weights were applied for complex sampling procedures. The characteristics of the study groups were described using frequencies and percentages**.**

The multiple multi-level logistic regression model was used to determine the association with factors. Due to the cluster’s nature, a generalised linear mixed model was fitted with a cluster-level random intercept.

We fitted four models. The first model was constructed without independent variables to assess the effect of community variation on UNFP among women. Individual-level factors were incorporated in the second model. In the third model, community-level factors were included. Finally, both individual-level and community-level factors were included in the analysis.

An adjusted Odds Ratio (AOR) with 95% CIs was computed to identify the independent factors of UNFP. A multicollinearity test was done to rule out a significant correlation between variables.

The random effects (variation of effects) were measured by intracluster correlation coefficient (ICC) (variance partition coefficient), the percentage change in variance (PCV), and median OR [36], which measure the variability between clusters in the multi-level models. ICC explains the cluster variability, while MOR can quantify unexplained cluster variability (heterogeneity). MOR translates cluster variance into the OR scale. In the multi-level model, PCV can measure the total variation due to factors at the community and individual levels.

A multi-level model provides correct parameter estimates by correcting the biases introduced from clustering by producing correct SEs, thus producing correct CI and significance tests [36–38]. All data processing and analysis were performed by using STATA version 14 software.

### Spatial analysis

Weighted prevalence was mapped to illustrate the distribution of unmet need in Ethiopia in ArcMap.

### Spatial autocorrelation analysis

The spatial autocorrelation (Global Moran’s I) statistic measures the unmet need patterns in the study area. A statistically significant Moran’s I (*p* < 0.05) was taken as an indicator of spatial autocorrelations.

### Hot spot analysis (Getis-OrdGi* statistic)

Hotspot Analysis (Getis-OrdGi*) statistics were computed to identify geographic areas that have significant high clusters and low clusters. The Z-score and *p*-value were computed for the significance. A high z-score and a small *p*-value (*p* < 0.05) for a feature indicate a high-value spatial clustering. Statistical output with high GI* indicates “hotspot”, whereas low GI* means a “cold spot”.

### Spatial interpolation

A Spatial interpolation technique was used to predict unmet need on the un-sampled areas in the country based on sampled EAs using Ordinary Kriging spatial interpolation methods.

### Spatial scan statistical analysis

Spatial scan statistical analysis was employed to test for the presence of statistically significant spatial clusters of unmet need using Kuldorff’s SaTScan version 9.6 software. The spatial scan statistic uses a circular scanning window that moves across the study area. Women with unmet need were taken as cases, and those who are no need for family planning as controls to fit the Bernoulli model. The numbers of cases in each location had Bernoulli distribution, and the model required data for cases, controls, and geographic coordinates. The default maximum spatial cluster size of < 50% of the population was used as an upper limit, which allowed both small and large clusters to be detected and ignored clusters that contained more than the maximum limit. For each potential cluster, a likelihood ratio test statistic was used to determine if the number of observed unmet needs within the potential cluster was significantly higher than expected or not. The primary, secondary and tertiary clusters were identified and assigned p-values and ranked based on their likelihood ratio test, based on 999 Monte Carlo replications.

### Operational definitions

#### Unmet need for family planning

Proportion of women who are not pregnant and not postpartum amenorrhoeic and are considered fecund and want to postpone their next birth for two or more years or stop childbearing altogether but are not using a contraceptive method, or have a mistimed or unwanted current pregnancy, or are postpartum amenorrhoeic and their last birth in the last two years was mistimed or unwanted. Percentage of fertile, sexually active women aged 15–49 who are not using contraception and do not wish to become pregnant (unmet need for limiting) or within the next two years (unmet need for spacing).

#### Met need for contraception

Number of women who are using a contraception method and are not considered limiting, want no more children, are sterilised, or say they cannot get pregnant when asked about the desire for future children.

#### Total demand for contraception

Number of women with a met need or unmet need: For spacing, for limiting, total.

#### Community women’s education

Was defined as the proportion of women who attended primary, secondary and higher education within the cluster. The aggregate of individual women’s primary, secondary, and higher educational attainment can show women’s overall educational status within the cluster. There were categorised into two categories as a higher proportion of women’s education within the cluster and a lower proportion of women’s education based on the national median value.

#### Community media exposure

Was defined as the proportion of women exposed to either television or radio within a cluster. The aggregate of individual women exposed to media and television can show women’s overall media exposure within the cluster. It was categorised into higher and lower community media exposure based on a national median value.

## Results

### Socio-demographic characteristics of women

A total of 15,683 women of reproductive age group were included in the final analysis, with 643 clusters nested in 11 regions. The mean (± standard deviation) age of the reproductive age group women was 28.2 (± 9.2). Overall, 9540(60.8%) women were in the age group of 25–49 years. The majority of the women, 12,207(77.8%), lived in a rural residence place. Nearly two-thirds, 10,223 (65.2%), of women were married or living with a partner. Almost half, 7498 (47.8%), of the women had no formal education. About one-third, 5442 (34.7%) of the women were from low-income households (Table 1).

**Table 1:** Socio-demographic characteristics of reproductive age women in Ethiopia, EDHS 2016(*n* = 15,683)

Variables	Weighted frequency	Weighted percent
Age of the respondents		
Mean ± SD of the women	28.2 ± 9.2	
15–19 years	3381	21.6
20–24 years	2762	17.6
25–49 years	9540	60.8
Place of Residence		
Urban	3476	22.2
Rural	12,207	77.8
Region		
Tigray	1129	7.2
Afar	128	0.8
Amhara	3714	23.7
Oromia	5701	36.4
Somali	460	2.9
Benishangul Gumuz	160	1.0
SNNP	3288	21.0
Gambela	44	0.3
Harari	39	0.3
Addis Ababa	930	5.8
Dire Dawa	90	0.6
Religion		
Orthodox Christian	6786	43.3
Muslim	4893	31.2
Protestant	3674	23.4
Catholic/tradition/others	330	2.1
Educational status of women		
No education	7498	47.8
Primary education	5490	35.0
Secondary education	1818	11.6
Higher Education	877	5.6
Marital status		
Never in union	4036	25.7
Married or in union	10,223	65.2
Separated/divorced/widowed	1423	9.1
Wealth status		
Rich	7263	46.3
Average	2978	19.0
Poor	5442	34.7
Total living children		
Less than five	11,890	75.8
Five or more	3793	24.2
Age at first marriage		
< 15 years	3055	19.5
15–24 years	8029	51.2
> 24 years	4599	29.3

### Women’s knowledge of contraceptive methods and source of information on family planning

Of the total women, almost all (98.3%) knew about any family planning methods. Only a quarter of women, 3890 (24.8%), were visited by family planning workers within the last 12 months. Overall, 6526(41.6%) of the women visited the health facility within the last 12 months. Nearly one-third, 4858 (31.0%), of the women were heard about family planning from radio, television, and magazines/news (Table 2).

**Table 2:** Variables related to women's knowledge of contraceptive methods andsource of information on family planning of women of reproductive age in Ethiopia, EDHS 2016(*n* = 15,683)

Variables	Weighted frequency	Weighted percent
Knowledge of any methods		
No	264	1.7
Yes	15,419	98.3
Visited by family planning workers within 12 months		
No	11,793	75.2
Yes	3890	24.8
Did the fieldworker talk about family planning		
No	1732	44.5
Yes	2158	55.5
Visited health facility within 12 months		
No	9157	58.4
Yes	6526	41.6
AT health facility, told about family planning		
No	4176	64.0
Yes	2350	36.0
Media exposure (Radio, TV, magazine/news)		
No	10,825	69.0
Yes	4858	31.0

### Prevalence of unmet need family planning

**Fig. 1:**
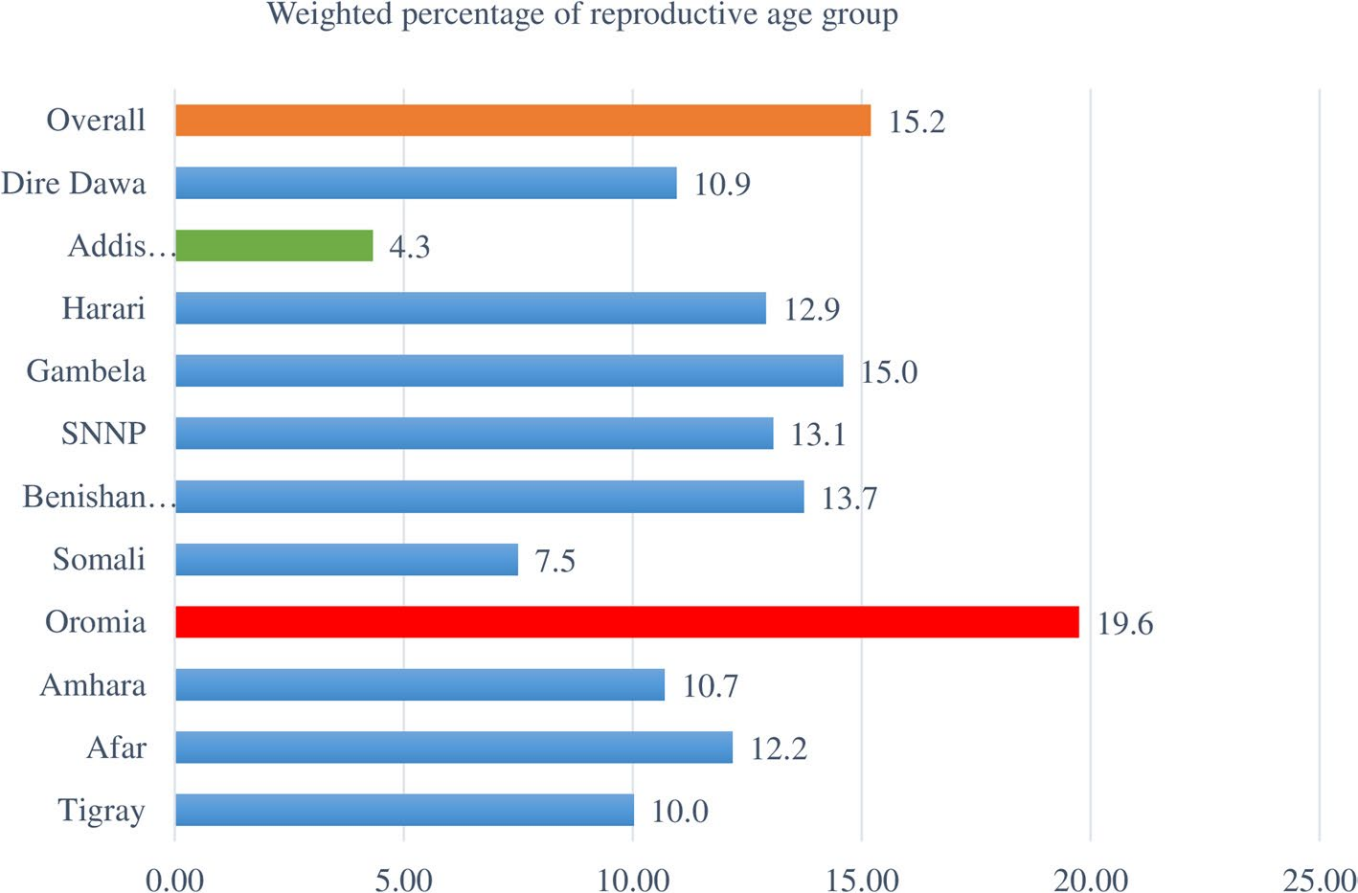
Percentage of reproductive-age women with unmet need for family planning by region, in Ethiopia, 2016

This study revealed that the magnitude of unmet need for family planning among reproductive-age women was 15.2% (95% CI 14.63, 15.76). The prevalence of unmet need for family planning among the urban and rural places of residence was 6.3% and 17.7%, respectively. There was a regional variation in the prevalence of unmet need for family planning among reproductive age group women in Ethiopia, 19.8% in the Oromia region while only 4.3% in Addis Ababa (Fig. 1).

### Factors associated with unmet need family planning

#### Multi-level logistic analysis

##### Random effects

About 20.2% of unmet need for family planning among reproductive-age women in clusters was explained in the full model, as calculated by ICC, so that it is good to fit the data by multi-level regression model as it is greater than 10%. The median odds ratio for unmet need for family planning was 1.63 in the full model, indicating a variation between clusters. This indicates that there was variation between clusters; hence median odds ratio was 1.63 in the empty model, which indicated that there was variation between communities (clustering) (63% times higher than the reference (MOR = 1)). If we randomly select women from two different clusters, women at the cluster with higher odds of unmet need had a 1.63 times higher chance of experiencing an unmet need for family planning than women at the cluster with lower risks of unmet need. The unexplained variation in unmet need increased when variables are added. This showed that the effect of clustering is still significant. About 54.5% of the variability in unmet need was explained by the full model (Table 3).

**Table 3:** Multivariable multilevel logistic regression analysis result of both individual and community-level factors associated with unmet need for family planning in Ethiopia, EDHS 2016

Individual and community level characteristics	Null model	Model II AOR (95% CI)	Model III AOR (95% CI)	Model IV AOR (95% CI)
Residence				
Urban			1	1
Rural			1.96 (1.41, 2.72)	1.37*(1.01,1.93)
Region				
Tigray			1	1
Amhara			0.87 (0.66, 1.16)	0.89 (0.66,1.20)
Afar			1.03 (0.74,1.42)	0.69 (0.45,1.05)
Oromia			1.79 (1.38,2.31)	1.53*(1.10,2.11)
Somali			0.55 (0.37,0.82)	0.37***(0.22,0.61)
Benishangul Gumuz			1.32 (1.01,1.73)	0.99 (0.73,1.37)
SNNPR			1.23 (0.93,1.61)	1.02 (0.74,1.40)
Gambella			1.90 (1.28,2.81)	1.94**(1.26,3.01)
Harari			1.69 (1.26,2.26)	1.26 (0.86,1.84)
Addis Ababa			0.87 (0.59,1.29)	1.19 (0.80,1.77)
Dire-Dawa			1.56 (1.07,2.28)	1.25 (0.80,1.96)
Age of respondents				
15–19 years		1		1
20–24 years		0.90 (1.10,1.74)		0.89 (0.62,1.28)
25–49 years		0.97 (0.69,1.35)		0.97 (0.69,1.36)
Marital status				
Never in union		1		1
Married or in unions		26.72 (11.55,61.83)		25.70***(11.50, 60.42)
Separated/divorced/widowed		1.51 (0.46,4.95)		1.55 (0.47,5.09)
Religion				
Orthodox		1		1
Muslim		1.38 (1.11,1.74)		1.35*(1.03,1.76)
Protestant		0.87 (0.67,1.13)		0.73*(0.54,0.99)
Catholic/traditions/others		2.08 (1.22, 3.54)		1.67 (0.96,2.91)
Wealth status				
Rich		1		1
Average		1.22 (0.96,1.55)		1.15 (0.90,1.48)
Poor		1.46 (1.18,1.81)		1.43**(1.14,1.79)
Women’s education				
No education		1		1
Primary education		1.17 (0.96,1.42)		1.16 (0.95, 1.42)
Secondary education		1.14 (0.76,1.70)		1.23 (0.82,1.86)
Higher education		1.20 (0.75,1.93)		1.37 (0.71,1.74)
Living children with the current pregnancy				
Less than five		1		1
Five and above		2.00 (1.65,2.43)		1.98***(1.62,2.41)
Age at first marriage				
Below 15 years		1		1
15–24 years		0.89 (0.74,1.07)		0.89 (0.74,1.07)
25 and above years		0.76 (0.46,1.24)		0.76 (0.46,1.24)
Knowledge of any FP methods				
No		1		1
Yes		2.28 (1.26,4.14)		1.56 (0.84,2.87)
Media exposure				
No		1		1
Yes		0.94 (0.76,1.16)		0.95 (0.77,1.17)
Visited by family planning workers within 12 months				
No		1		1
Yes		0.98 (0.84,1.15)		0.96 (0.83,1.14)
Visited health facility within 12 months				
No		1		1
Yes		0.92 (0.76,1.01)		0.92 (0.77,1.11)
Community-level education				
Low			1	1
High			0.85 (0.69,1.04)	0.90 (0.72,1.14)
Community-level media exposure				
Low			1	1
High			0.69 (0.55,0.85)	0.98 (0.79,1.21)
Random effects				
ICC	35.8	24.9	23.3	20.3
Log-likelihood (LL)	-6021.24	-5160.85	-5938.67	-5126.52
Deviance(-2LL)	12,042.48	10,321.70	11,877.34	10,253.04
PCV-Explained variation	Ref	40.5	46.6	54.3
MOR	2.07	1.75	1.69	1.62

Key: COR Crude odds ratio AOR: Adjusted odds ratio; *CI* Confidence interval, ICC; 1: reference group; *p*-value 0.05–0.01 *: *P*-value < 0.01 **: *p*-value < 0.001***

##### Fixed effects

Multivariable Multi-level logistic regression analysis of individual-level factors associated with unmet need was religion, wealth status, marital status, and the total number of children. In contrast, community-level factors, place of residence, and region were significantly associated with unmet need.

After controlling for potential confounders in the multivariable analysis revealed that the odds of having unmet need among women who lived in a rural place of residence was higher by 37% compared to urban dwellers (AOR = 1.37, 95% CI:1.01,1.93), the likelihood of unmet need among married/in union women was about 25.7 times more common compared to those never in union women (AOR = 25.7, 95% CI: 11.50, 60.42), the odds of having unmet need among women who had five or more living children was higher by 98% compared to those women who had less than five children (AOR = 1.98, 95% CI: 1.62,2.41), the odds of having unmet need among women in lower wealth quintile was higher by 43% (AOR = 1.43, 95% CI: 1.14,1.79) compared to those in lowest wealth, the odds of having unmet need among Muslim religion followers was higher by 35% compared to those women who are Orthodox religion followers (AOR = 1.35,95% CI: 1.03,1.76). However, the odds of having unmet need among protestant religion followers were lower by 27% compared to orthodox Christian followers (AOR = 0.73,95% CI:0.54,0.99), the odds of having unmet need among women in Addis Ababa, Amhara, Afar, SNNPR, Benishangul-Gumuz, Harari, and Dire Dawa was not significantly different from those having unmet in Tigray. The odds of having unmet need among women in the Oromia region was higher by 53% compared to women in the Tigray region (AOR = 1.53, 95%CI: 1.10, 2.11), and the odds of having unmet need among women in the Gambella region was higher by 94% compared to women in Tigray region (AOR = 1.94, 95%CI: 1.26, 3.01). However, the odds of having unmet need among women in the Somali region were lower by 63% than women in the Tigray region (AOR = 0.37, 95%CI: 0.22, 0.61) (Table 3).

##### Spatial distribution of unmet need for family planning

**Fig. 2:**
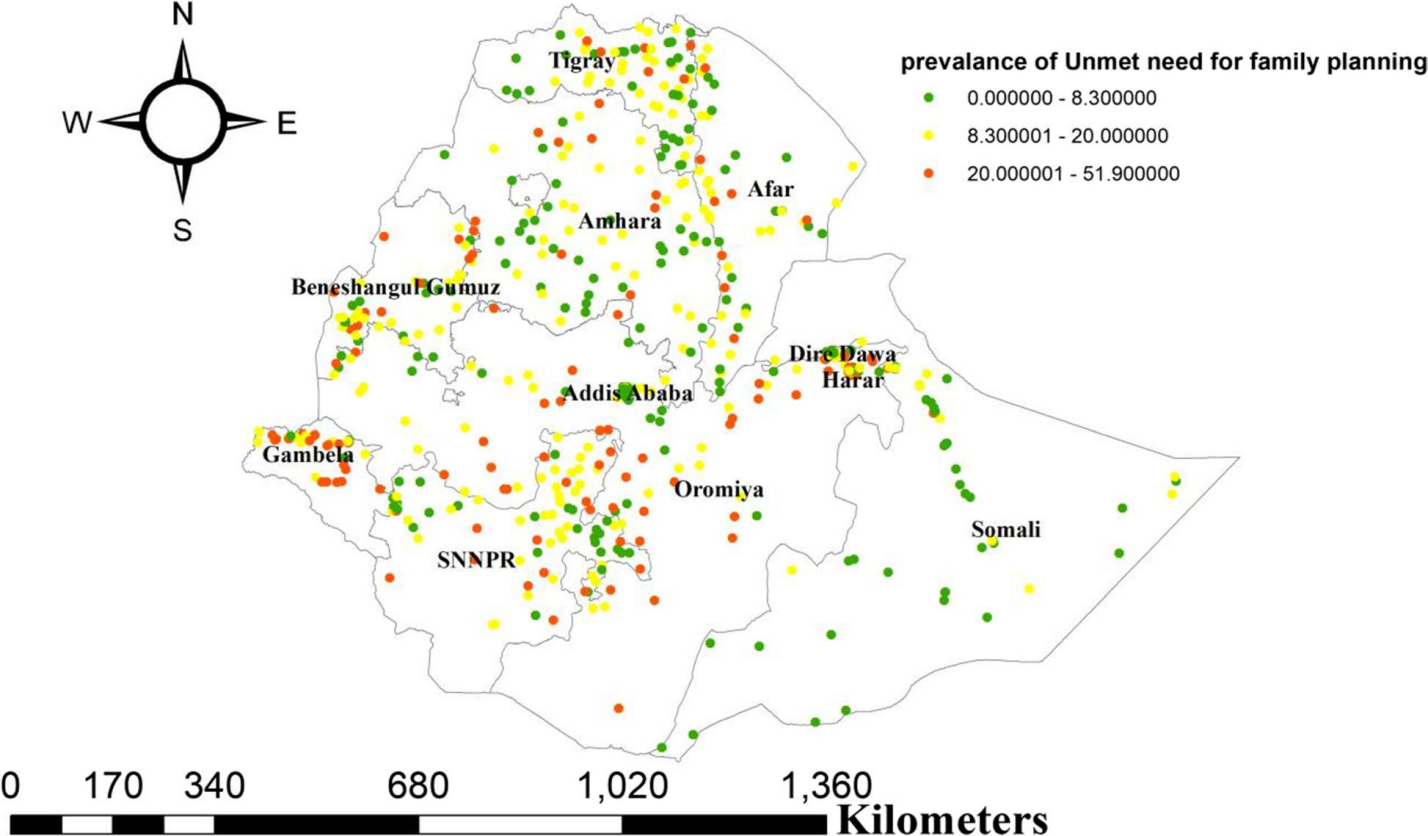
Spatial distribution of unmet need family planning among reproductive age group women in Ethiopia, 2016

About 622 clusters were considered for spatial analysis of the unmet need for family planning. A higher proportion of unmet need occurred in Northeast Tigray, East Afar, Northwest Amhara, South of Benishangul-Gumuz, Northeast of SNNP, North of Oromia region, Dire-Dawa, Harari, and Gambella (Fig. 2). The Spatial autocorrelation analysis revealed that the distribution of unmet need for family planning was non-random in Ethiopia with Global Moran’s I of 0.28 (*p*- value < 0.001).

##### Hot spot analysis of UNFP

**Fig. 3:**
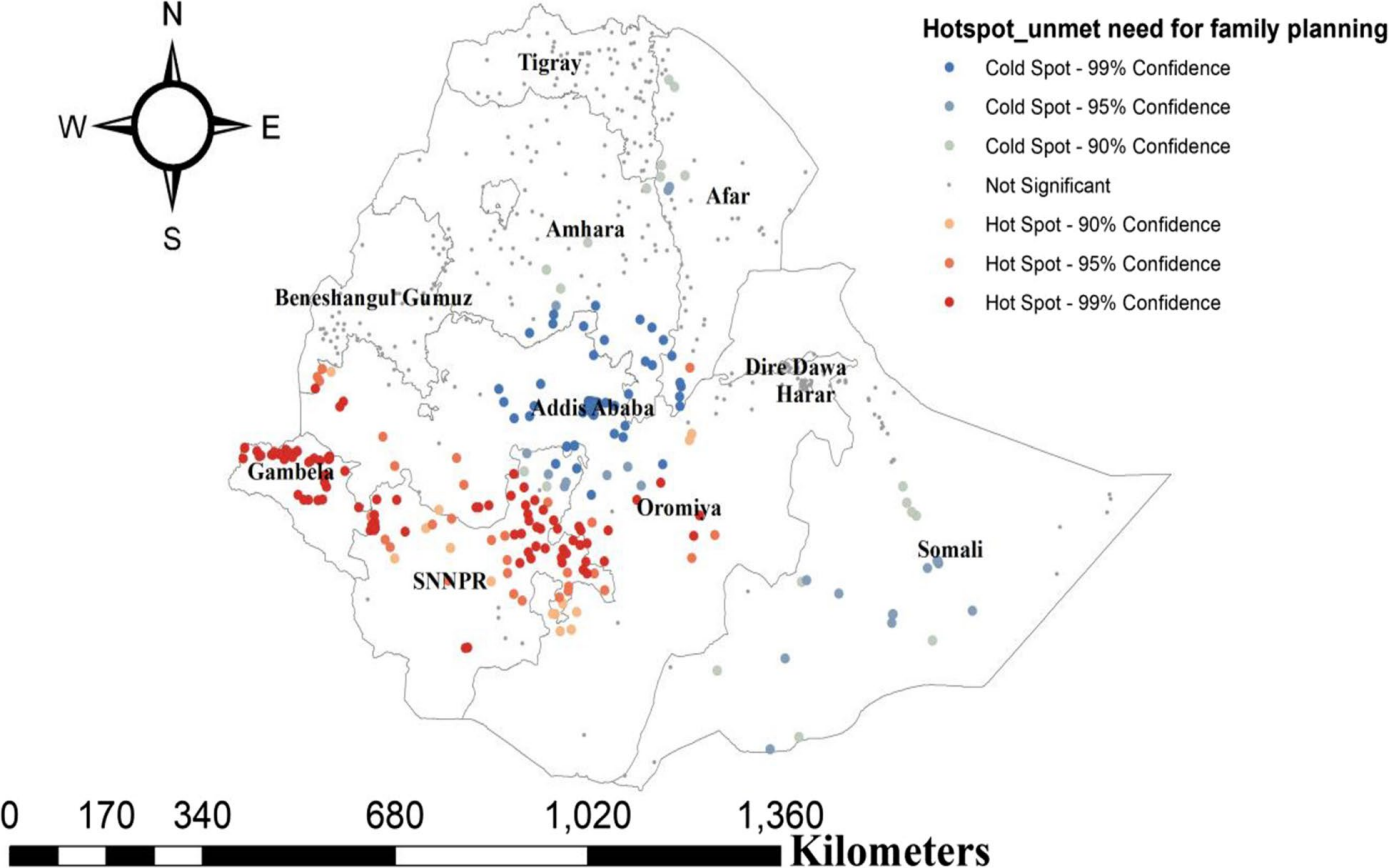
Hot spot analysis of unmet need family planning among reproductive age group women in Ethiopia, 2016

The significant risky areas (high rate of unmet need) were found in Gambela, Oromia, and SNNP regions of Ethiopia (*p*-value < 0.01 (Fig. 3). Hot spot areas of unmet need for family planning were found in Northwest Amhara, Gambela, and Northern parts of SNNP, Northern parts of Oromia, and Dire-Dawa regions, while cold spot areas were found in Southeast Tigray, Southern Amhara, Addis Ababa, and Somali regions. Outliers were found in Southwest Tigray, Addis Ababa, Northwest Afar, Harari, and Dire-Dawa.

### Interpolation of unmet need for family planning

**Fig. 4:**
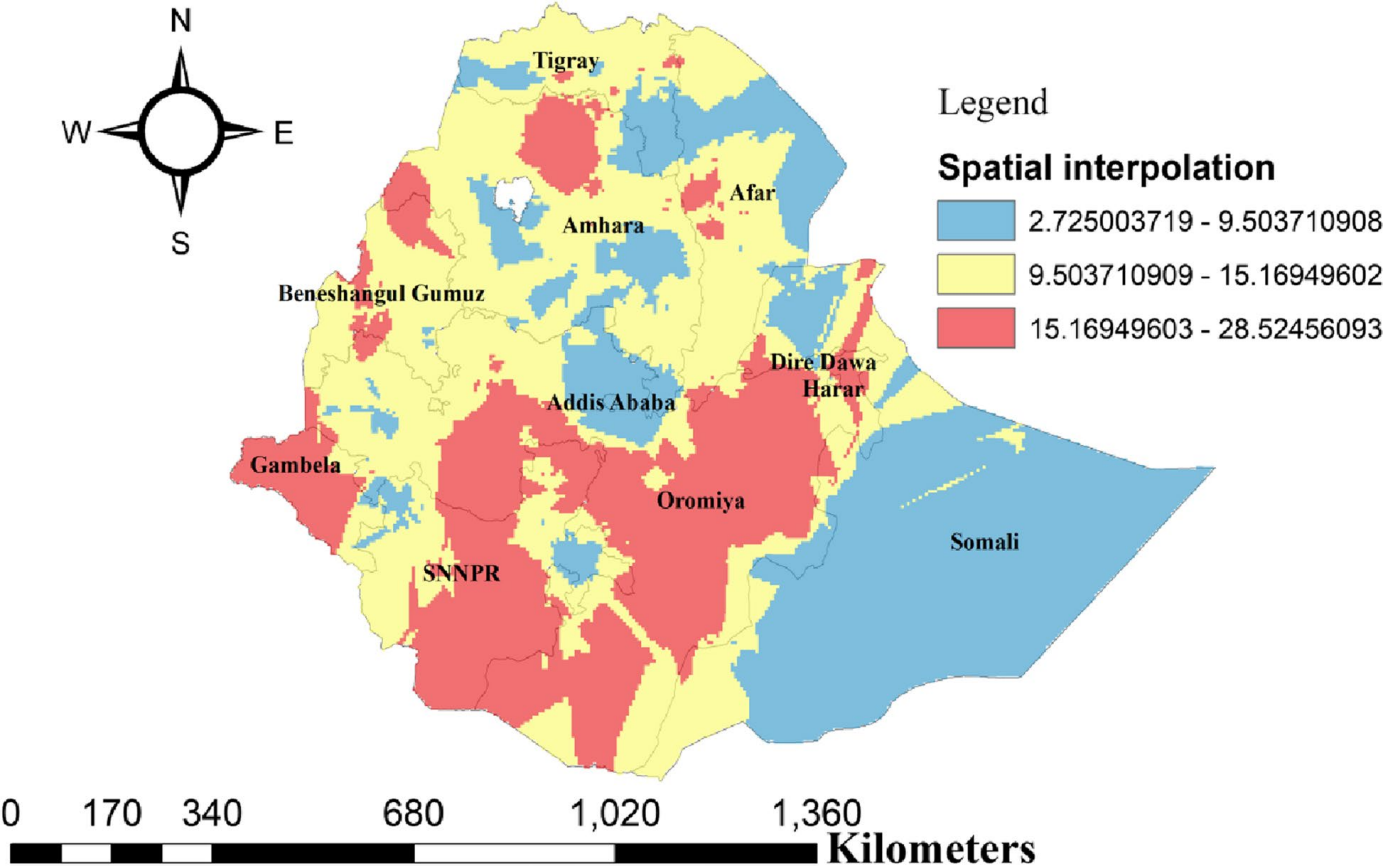
Interpolation of unmet need family planning among reproductive age group women in Ethiopia, 2016

Women in Gambela, Northwest Amhara, Northern and Northwest Oromia, and Northern SNNP regions are predicted to have a more unmet need for family planning than women residing in other areas. (Fig. 4).

### Spatial SaTScan analysis of the unmet need for family planning

Most likely (primary clusters), secondary clusters, and tertiary clusters of unmet need were identified. A total of 149 significant clusters were identified. Of these, 122 were most likely (primary), 19 secondary, and 8 tertiary clusters.

**Fig. 5:**
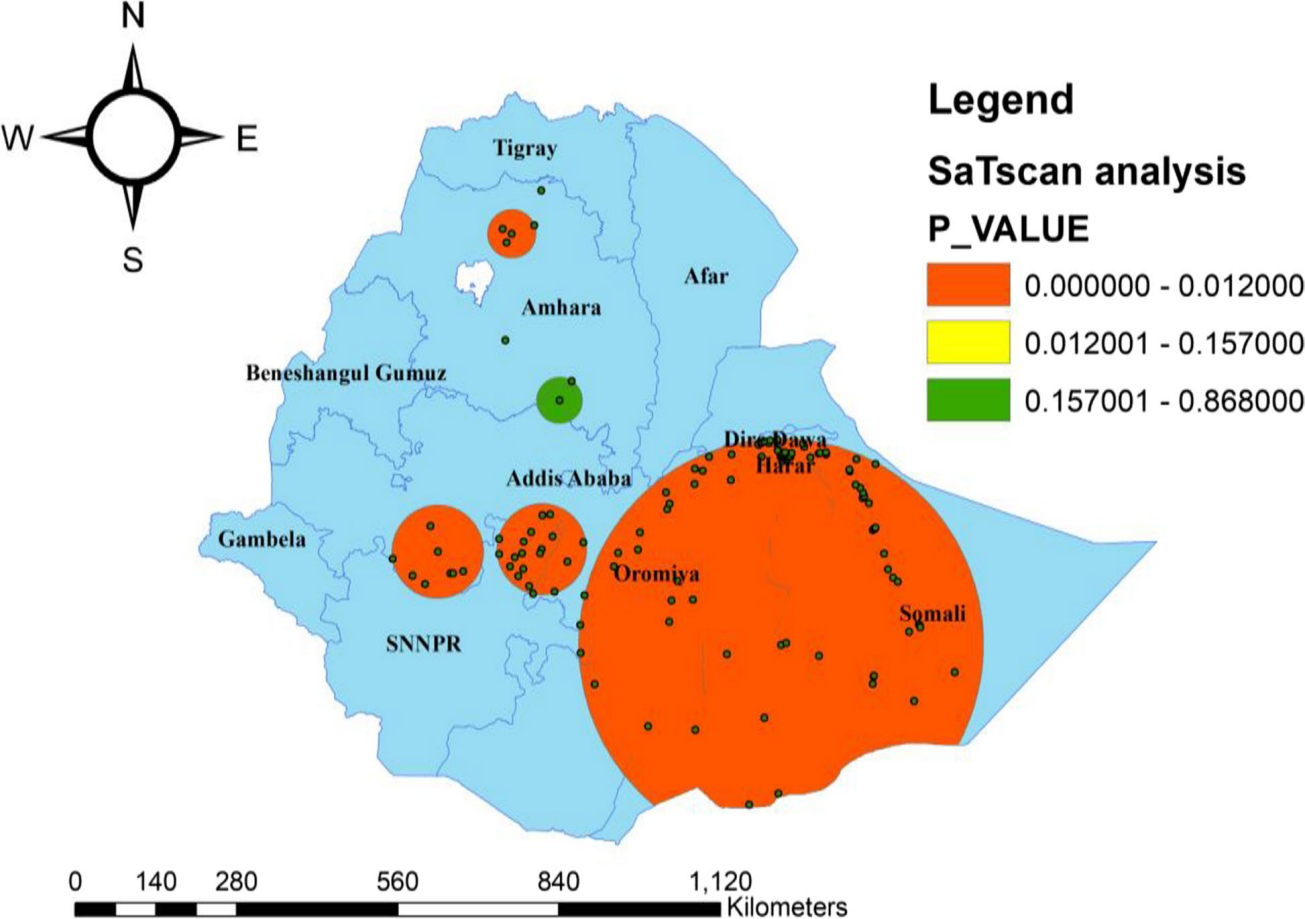
SaTScan analysis of the unmet need for family planning among all women in the last five years in Ethiopia, 2016

The primary clusters’ spatial window was located in Somali, Eastern Oromia, and Harari regions (Log-Likelihood ratio (LLR) = 85.16, *p*-value < 0.001). It showed that women within the spatial window had 1.88 times higher risk of unmet needs than women outside the window. The secondary clusters’ spatial window was typically located in the Western part of the Oromia region (LLR = 37.24, *p*-value < 0.001). It showed that women within the spatial window had a1.82 times higher risk of unmet needs than women outside the window. The tertiary clusters’ spatial window was typically located in the Western part of the Oromia region and Northern SNNP (LLR = 16.71, *p*-value < 0.001). It showed that women within the spatial window had 1.66 times higher risk of unmet needs than women outside the window (Table 4, Fig. 5).

**Table 4:** SaT Scan analysis of the unmet need for family planning among reproductive age group women within the last five years in Ethiopia, 2016

Cluster	Coordinate/radius	No_ of Clusters	population	cases	Expected cases	RR	LLR	*P*-value
Primary	(6.441558 N, 42.095158 E) / 348.82 km	122	2361	541	327	1.88	85.16	< 0.001
Secondary	(7.896075 N, 38.358570 E) / 77.01 km	19	941	226	130	1.82	37.24	< 0.001
Tertiary	(7.858150 N, 36.733551 E) / 78.67 km	8	603	135	84	1.66	16.71	< 0.001

## Discussion

In this study, about 15% of all reproductive age group women in Ethiopia had an unmet need for family planning. It showed that these women’s unmet needs were more petite than those who were only currently married women. This might be because married women had more sexual exposure than all other women. This difference might also be due to an increase in denominators of unmet need calculation for all reproductive age groups than only currently married women. The current finding was also lower than a national survey in Uganda among all women. This finding might be due to the study time difference, a study in Uganda that was 7 years earlier than the current study. During this study period, there was a difference in awareness of the unmet need for family planning and a change in the operational definition [3, 39].

According to the final model, both individual-level and community-level factors were responsible for approximately 55% of the unmet need prevalence rates among all reproductive-age group women in Ethiopia. After adjusting for all factors in the model, the likelihood of experiencing unmet needs was higher among those living in rural areas, in the lowest wealth quintile, who were currently married or in the union, with more than five living children, who were Muslim religion followers and who were from Oromia and Gambela regions. However, women were protestant religious followers and in Somali regions had a lower likelihood of the unmet need for family planning.

The current study revealed that the likelihood of the unmet need for those women who had more than five living children was 98% times more likely compared to those having fewer children. This finding supports previous studies done in Ethiopia, Burkina Faso, and India that showed a positive association between unmet needs and more children [12, 15, 19]. This difference might be because when women have more children, they want to limit and space birth due to physical, social, and economic consequences followed by births.

This study also showed that women in poor, wealth-quintile households were 43% more likely to unmet needs than women who belong to the affluent quintile. This finding is consistent with the study results in Ghana (AOR:2.0:1.36–2.96)[18]. This might be because having a low economic status would mean having less money for transportation to the health facility to utilise family planning. It could also be a lack of access to health services due to economic reasons (economic barriers) or inadequate family planning knowledge.

The religion of the respondents was significantly associated with the unmet need for family planning. Muslim religious follower women were 35% more likely to have an unmet need for family planning than those Orthodox religious followers. This finding supports the positive association between Muslim religious followers and the unmet need for family planning in India [14]. To put the reason for the discrepancy between religions is ambiguous but it might be religious prohibition leads women to have many children.

Furthermore, this current study revealed a significantly substantial difference in the proportion of unmet needs according to the place of residence (urban/rural). The likelihood of having UNFP was 37% higher for rural residents compared with urban residents. This finding agreed with a study conducted in Ethiopia’s different regions [12, 23, 33]. These higher unmet needs in rural areas might be due to limited awareness and lower educational status prohibiting the utilisation of family planning services. A high unmet need for family planning in rural areas might be due to inadequate access to health services or inadequate family planning awareness.

Our study findings revealed that women from Oromia and Gambela regions were more likely to have an unmet need for family planning. However, women from the Somali region were less to have unmet needs compared to the Tigray region. The possible justification in these regions might be that infrastructures did not satisfy the women’s family planning demand. Another possible explanation for this difference might be due to the sociocultural difference between the two regions. The low demand for family planning in the Somali region (10%) vs the Tigray region (36.2%) could also be associated with a low rate of premarital sexual activity. The spatial distribution of unmet needs in family planning supports this finding among all reproductive age group women in our study.

The current study revealed significant geographical variations among regions in Ethiopia, particularly the regions of Oromia and Gambela had higher prevalence rates than other regions. The populations of the two regions might have an inadequate family planning infrastructure. The predicted unmet need showed that Gambela, Northwest Amhara, northern and Northwest Oromia, and northern SNNP were predicted as riskier than other regions. This might be the relative underdevelopment and inadequate infrastructure that may contribute to the inadequate family planning service.

### Strengths and limitations

We used extensive population-based data with large sample size, representing all regions of Ethiopia. Furthermore, a combination of statistical methods (spatial analysis and multi-level logistics analysis) was applied for this study to understand the role of contextual and geographical factors in the occurrence of unmet needs among women of reproductive age. Due to the EDHS data’s cross-sectional nature, the cause/effect and the temporal relationship could not be established based on these study findings. Ethiopian demographic and health survey were questionnaire-based survey and relied on the respondents’ memory, and as such, recall bias in the results might be a weakness for this study.

## Conclusion

The prevalence of unmet needs for family planning among all reproductive age group women showed a significant improvement compared to 2011 EDHS. Findings in the current study also showed that UNFP was higher among women who lived in rural areas, from in the lowest wealth quintile, were married, had more children, were Muslim religion followers, and were from Oromia and Gambela regions.

The spatial analysis also revealed that unmet needs among women varied across regions in the country; significant unmet need rate hotspots were generally observed in Gambela, Northern SNNPR, and the Oromia region.

A decrement in unmet needs among women requires multifaceted intervention approaches, for instance, increasing trained family planning workers, construction of roads, and health facilities to access family planning services for poor and rural women with strengthening the HEWs and HDAs. However, many more factors could contribute to the high unmet need in rural settings. As a result, further studies shall be conducted for this specific population group to identify concrete evidence. The Ethiopian government should prioritise Gambela and Oromia regions that are unmet needs for family planning hotspots.

## Data Availability

The data sets used during the current study are available from the corresponding author on a reasonable quest.

## Abbreviations

AIC: Akakies information criteria
AOR: Adjusted odds ratio
BIC: Bayesian information criteria
CI: Confidence Interval
COR: Crude odds ratio
DHS: Demographic Health Survey
EDHS: Ethiopian Demographic and Health Survey
FMOH: Federal Ministry of Health
GLMM: Generalised linear mixed models
HAD: Health Development Army
HEWs: Health Extension workers
ICC: Intra-cluster correlation coefficient
MOR: Median odds ratio
PCV: Proportional Change in Variance
PHC: Population and Health Survey
SNNP: Southern Nations and Nationality of People
UNFPA: United Nations Fund for Population Activities
